# Valorizing Organic Waste: Selenium Sulfide Production Mediated by Sulfate-Reducing Bacteria

**DOI:** 10.3390/ma18122784

**Published:** 2025-06-13

**Authors:** Shahrzad Safinazlou, Ahmad Yaman Abdin, Eduard Tiganescu, Rainer Lilischkis, Karl-Herbert Schäfer, Claudia Fink-Straube, Muhammad Jawad Nasim, Claus Jacob

**Affiliations:** 1 Division of Bioorganic Chemistry, School of Pharmacy, Saarland University, 66123 Saarbruecken, Germany; shsa00006@uni-saarland.de (S.S.); yaman.abdin@uni-saarland.de (A.Y.A.); s9edtiga@stud.uni-saarland.de (E.T.); 2 Working Group Informatic and Microsystems Technology Department, University of Applied Sciences Kaiserslautern, Amerikastrasse 1, 66482 Kaiserslautern, Germany; 3 Working Group Enteric Nervous Systems (AGENS), University of Applied Sciences Kaiserslautern, Amerikastrasse 1, 66482 Kaiserslautern, Germany; karlherbert.schaefer@hs-kl.de; 4 INM—Leibniz Institute for New Materials, 66123 Saarbrücken, Germany; claudia.fink-straube@leibniz-inm.de

**Keywords:** cabbage juice, compost, hydrogen sulfide, milk, mineral water, selenium sulfide, silage, sulfate-reducing bacteria, waste-to-value

## Abstract

Selenium sulfide, the active ingredient of traditional antidandruff shampoos, is industrially produced from selenium dioxide (SeO_2_) and hydrogen sulfide (H_2_S) under acidic conditions. This reaction can also be carried out with natural H_2_S and H_2_S generated by sulfate-reducing bacteria (SRB). These bacteria are robust and, by relying on their conventional growth medium, also thrive in “waste” materials, such as a mixture of cabbage juice and compost on the one side, and a mixture of spoiled milk and mineral water on the other. In these mixtures, SRB are able to utilize the DL-lactate and sulfate (SO_4_^2−^) present naturally and produce up to 4.1 mM concentrations of H_2_S in the gas phase above a standard culture medium. This gas subsequently escapes the fermentation vessel and can be collected and reacted with SeO_2_ in a separate compartment, where it yields, for instance, pure selenium sulfide, therefore avoiding the need for any cumbersome workup or purification procedures. Thus “harvesting” H_2_S and similar (bio-)gases produced by the fermentation of organic waste materials by suitable microorganisms provides an elegant avenue to turn dirty waste into valuable clean chemical products of considerable industrial and pharmaceutical interest.

## 1. Introduction

Recent years have witnessed an increased interest in developing sustainable green approaches to produce molecules relevant for pharmaceutical and cosmetic industries [1,2,3,4]. Whilst in many cases traditional methods remain unavoidable to meet strict production and quality standards, they tend to be resource and energy intensive. Alternative, more sustainable and green approaches promise to reduce this demand for resources, the environmental impact and costs associated with the production of such molecules and the notion of turning “waste products” into valuable compounds, possibly with the aid of low-energy fermentation processes, are especially attractive for several of those reasons [5,6,7].

We have recently turned our attention to the production of selenium sulfide. The conventional synthesis involves the reduction of Se^4^⁺ from selenium dioxide (SeO_2_) by sulfide ions (S^2−^) from sodium sulfide (Na_2_S), leading to the formation of elemental selenium (Se^0^), which subsequently reacts with additional S^2−^ to form polymeric selenium sulfide (Se_n_S_8-n_). The reaction proceeds via nucleophilic attack and redox coupling, resulting in a precipitate of Se–S compounds. The reaction takes place in the presence of glacial acetic acid, which supports the precipitation of selenium sulfide. Once the reaction is completed, the product is separated by centrifugation, washed with water and dried to obtain the desired compound [8,9,10,11]. Whereas SeO_2_ is not easily available outside the mining and chemical industry, H_2_S is an abundant natural product found, for instance, in volcanic and mineral waters, and may therefore be harvested from a sustainable, green source [12,13,14,15]. And indeed, it has been possible to perform the synthesis of selenium sulfide with natural spring water from the spring of Bad Nenndorf in Lower Saxony, Germany, as reported by us in this journal [16,17]. Although this approach, based on a natural, inorganic H_2_S source is very promising, it also has its limitations. The geographical distribution of and access to these wells is often limited, and this may require complex logistics and the transport of waters across longer distances. Furthermore, the concentrations of H_2_S in these waters are modest, and this, in turn, requires the handling of considerable amounts/volumes of water and extensive filtration to harvest the selenium sulfide produced [16].

We have therefore followed up this initial approach and turned our attention to other, more readily available organic sources of H_2_S. Inspired by the fact that H_2_S often occurs in high concentrations in cesspits, the microbial fermentation of organic materials and especially organic waste may provide an alternative, more convenient access to this gas [18,19]. Indeed, sulfate-reducing bacteria (SRB) reduce sulfate (SO_4_^2−^) to H_2_S through the dissimilatory sulfate reduction (DSR) pathway where SO_4_^2−^ serves as a terminal electron acceptor [20,21]. Reduction of SO_4_^2−^ to H_2_S involves several intermediates and the process of DSR requires the transfer of eight electrons in total [22,23]. In contrast to the assimilatory sulfate reduction pathway, which incorporates sulfate into sulfur-containing amino acids and proteins, the dissimilatory pathway produces H_2_S as a stable product which can be readily collected for synthetic applications, offering a more amenable approach [24,25]. Notably, several of these SRB rely on DL-lactate as carbon source, and since lactate is found naturally in many types of agricultural and food wastes, such as silage and spoiled milk, this strategy indeed has attracted our immediate attention [26,27,28,29].

Here, we report our first investigations of the selenium sulfide synthesis with H_2_S produced from organic sources and naturally occurring mineral SO_4_^2−^ sources. Among the organic sources are common, highly abundant, readily available and low-cost waste materials, including the fermentation juice of vegetables, spoiled milk and compost, whereas mineral water from gypsum-rich soils may serve as natural source for SO_4_^2−^.

It should be emphasized from the beginning that to turn these–often dirty–waste materials into the value of a clean chemical product is a considerable challenge, especially if the methods employed need to be “green”, (cost) effective, low energy, and suitable for large-scale processing avoiding extensive purification. Thus, a special and sophisticated sequence of biological and chemical processes had to be devised in order to meet these various challenges, and although the ensuing strategy presented here may look a little “redneck” at first, its design is far from trivial.

It should also be noted that this is an initial feasibility study and that optimization of the various processes is, of course, always possible yet needs to be the topic of a further follow-on manuscript.

## 2. Materials and Methods

### 2.1. Materials

Chemicals were purchased from Merck (Darmstadt, Germany) unless stated otherwise. *Desulfovibrio desulfuricans* (DSM 642) was purchased from the Leibniz Institute DSMZ–German Collection of Microorganisms and Cell Cultures GmbH (Braunschweig, Germany) [30]. Amongst various compost compositions available commercially, “Bio Hochbeet Kompost 2 torffrei” was selected and purchased from a local retail store, Bauhaus (Saarbruecken, Germany), at a cost of less than three Euros per 10 kg. Fermented cabbage juice (Alnatura Sauerkraut Saft), kefir (Kalinka Kefir (low fat)) and low-fat milk are available in shops and thus were purchased from local supermarkets such as Netto and Müller (Saarbruecken, Germany). The same products can be collected for free once they have met their expiration date. Ensinger Schiller Quelle mineral water, used as a mineral water source, was purchased from the Alldrink local beverage store in Saarbruecken, Germany. Plastic fermentation tubes (airlock for trapping H_2_S) were purchased from Amazon. Grass silage was collected from the Bannsteinhof farm in Zweibruecken, Rhineland-Palatinate, Germany (49°14′56.4″ N, 07°21′53.6″ E) in August 2024. Red clover was harvested from Saarland University, Saarbruecken, Germany (49°15′32.0″ N, 07°02′25.4″ E in October 2024 and identified as *Trifolium pratense* using the PlantApp application (Scale Up, Izmir, Turkey) [31]. Mifloran intense containing *Bifidobacterium* and *Lactobacillus* species used for silage fermentation were purchased from the local Reformhaus store in Saarbruecken, Germany.

### 2.2. Design of Fermentation Equipment

**Figure 1:**
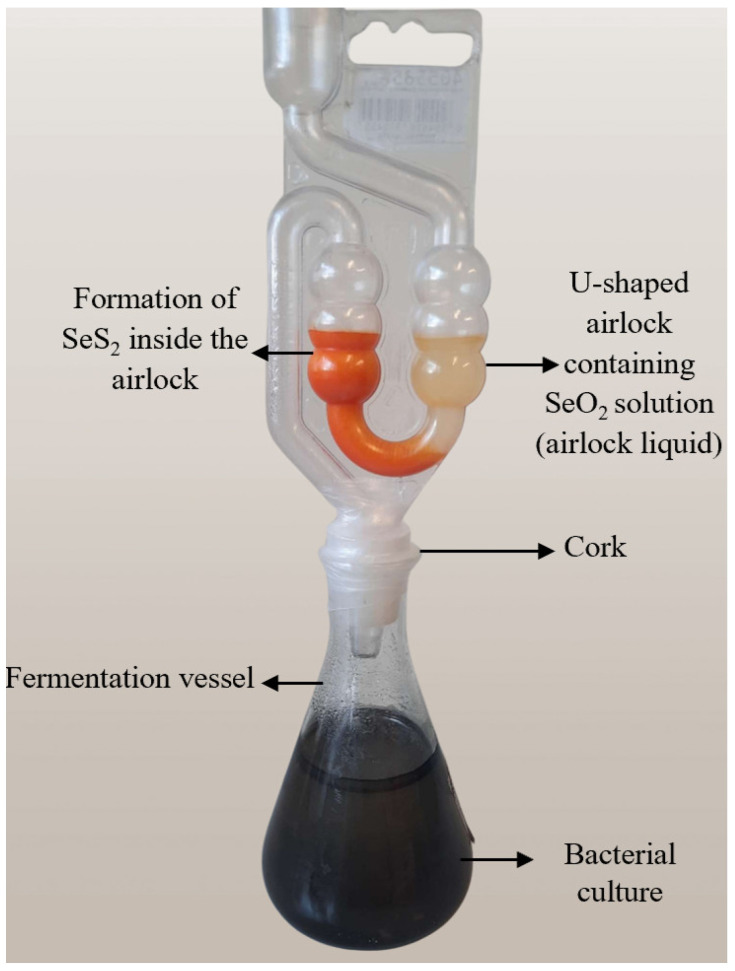
SRB were incubated under anaerobic conditions in specially designed fermentation equipment, including a fermentation vessel coupled to fermentation tube (airlock). This photo of actual SRB fermentation equipment is provided by the first author (SS).

The design of the fermentation equipment is shown in Figure 1. It consists of a standard fermentation vessel enabling the anaerobic fermentation with SRB and the collection of the gasses produced in a U-shaped airlock, which at the same time serves as a reaction vessel for the reaction of H_2_S with SeO_2_.

### 2.3. Cultivation of SRB

*D. desulfuricans* is a SO_4_^2−^ reducing anaerobic bacterium which was cultured according to the special instructions provided by the Leibniz Institute DSMZ–German Collection of Microorganisms and Cell Cultures GmbH (Braunschweig, Germany) for handling the anaerobic bacteria [30]. In brief, the standard cultivation medium comprises three different solutions, i.e., solutions A, B and C. Solution A consisted of K_2_HPO_4_ (0.5 g), NH_4_Cl (1.0 g), Na_2_SO_4_ (1.0 g), CaCl_2_•2H_2_O (0.1 g), MgSO_4_•7H_2_O (2.0 g), sodium DL-lactate (2.0 g), yeast extract (1.0 g) and sodium resazurin (0.1% *w*/*v*, 0.5 mL), dissolved in 980 mL of distilled water. Solution B consisted of a solution of FeSO_4_•7H_2_O (0.5 g) dissolved in 10 mL of distilled water and solution C contained sodium thioglycolate (0.1 g) and ascorbic acid (0.1 g) dissolved in 10 mL of distilled water. Solution A was boiled for 5 min and subsequently cooled to room temperature under a continuous flow of nitrogen gas to maintain anaerobic conditions. This step was followed by the addition of solutions B and C. Lastly, the pH of the solution was adjusted to 7.0 using a few drops of NaOH and maintained without further dynamic adjustment during incubation. After autoclaving, the medium was carefully transferred to the fermenting equipment under anaerobic conditions, followed by the inoculation of the bacteria. The cultures were incubated at 30 °C in the Environmental Shaker–Incubator ES20 toprovide, at stable temperature and agitation throughout the fermentation process (Grant Instruments, Royston, UK).

The composition of the medium was later optimized to find the essential ingredients required for the cultivation of bacteria and the production of H_2_S as shown in the Supplementary Section, Table S1. Then, the essential ingredients of the medium were gradually replaced with more common “household” ingredients and eventually waste products, i.e., silage, cabbage juice and spoiled milk were used as a substitute for DL-lactate while compost and mineral water were added as a source of SO_4_^2−^ and minerals (summarized in Table S2 in Supplementary Materials).

### 2.4. Quantification of DL-Lactic Acid

DL-lactic acid, a key component of the standard medium, serves as a carbon source for bacterial growth and the synthesis of essential cellular structures. Thus, the initial step during replacement with silage, spoiled milk, etc., involved quantifying the concentration of DL-lactic acid in commercially available fermented cabbage juice and kefir obtained from local supermarkets, as well as monitoring its concentration over time in homemade red clover silage and spoiled milk. The quantification of DL-lactic acid was carried out using the protocol described in the literature [32]. Briefly, the standard curve was prepared employing different concentrations of DL-lactic acid ranging from 0 to 300 mg L^−1^. Solutions containing 100 µL of DL-lactic acid were diluted with 400 µL of distilled water followed by the addition of 3 mL H_2_SO_4_ (95%). The reaction mixtures were heated at 95–100 °C for 10 min and then cooled to room temperature, followed by the addition of CuSO_4_•5H_2_O solution (50 µL, 4% *w*/*v*) and *p*-phenylphenol solution (100 µL, 1.5% *w*/*v* in 95% ethanol). After mixing, the resultant acetaldehyde-*p*-hydroxyphenyl complex was incubated at room temperature for 30 min and the absorbance was recorded at 570 nm using a BioTek Epoch 2 Microplate Spectrophotometer (BioTek Instruments GmbH, Bad Friedrichshall, Germany). A similar procedure was performed for milk and red clover samples. Briefly, samples were prepared as follows: 300 mL milk samples in tightly closed containers were used for measuring their DL-lactic acid content. Red clover samples were collected and chopped into small pieces, divided and stored in small tightly sealed glass containers, each containing approximately 16 g of red clover. Subsequently, the samples were inoculated with 100 µL culture containing *Bifidobacterium* and *Lactobacillus* species to promote fermentation at room temperature. Experiments were conducted in triplicate, with three subsamples obtained from each replicate (*n* = 9). Results are presented as arithmetic means.

### 2.5. Quantification of H_2_S

Following the preparation and inoculation of the culture media, the SRB initiated H_2_S production, which accumulated in the headspace of the fermentation vessel and dissolved in the solution within the airlock. H_2_S was collected in the headspace (40 mL) of an Erlenmeyer flask (115 mL) containing bacterial culture (75 mL). The headspace-to-liquid ratio of approximately 0.5 was maintained to ensure sufficient space for gas accumulation without excessive pressurization. The quantification of H_2_S in the headspace was performed employing the methylene blue (MB) assay as described in the literature, with a few modifications [33]. The standard curve was prepared using different concentrations of anhydrous sodium sulfide (Na_2_S) ranging from 0 to 0.6 mM. An amount of 100 µL from the gas phase (headspace) was removed with a syringe and mixed with 1 mL FeCl_3_•6H_2_O (30 mM) dissolved in HCl (1.2 M) and 1 mL *N*,*N*-dimethyl-*p*-phenylenediamine dihydrochloride (DMPD, 20 mM) dissolved in HCl (7.2 M). The reaction mixture was incubated at room temperature for 15 min, and the absorbance was recorded at 670 nm using a microplate spectrophotometer. Experiments were in triplicate, with three subsamples from each replicate (*n* = 9). Results are presented as arithmetic means.

### 2.6. Production of Selenium Sulfide

The reaction of H_2_S with SeO_2_ solution (100 mM) was carried out in the airlock directly attached to the incubation assembly as shown above in Figure 1. The samples of selenium sulfide were collected from the airlock after 310 h of incubation as the production of H_2_S was completed by then and stabilized at baseline level. The selenium sulfide suspension was centrifuged at 3000 rpm for 15 min. The supernatant was discarded subsequently, and the pellet was washed with distilled water (50 mL) three times to eliminate unreacted SeO_2_. The collected selenium sulfide was dried and stored in the dark at room temperature until further use.

### 2.7. Characterization of Selenium Sulfide

The selenium sulfide obtained was characterized using analytical techniques, such as CHNS analysis, Scanning Electron Microscopy with Energy Dispersive X-ray Spectroscopy (SEM-EDX), Raman spectroscopy and Optical Emission Spectrometry combined with Inductively Coupled Plasma (ICP-OES). CHNS analysis was carried out employing a Vario MICRO cube CHN-elemental analyzer (Elementar GmbH, Langenselbold, Germany). SEM-EDX analysis was conducted with a ZEISS Supra 40 field emitter microscope (Carl Zeiss NTS GmbH, Oberkochen, Germany) coupled to a Bruker Quantax EDX system (Bruker Nano GmbH, Berlin, Germany). Raman spectroscopy was performed on a Renishaw InVia microscope (Wotton-under-Edge, Gloucestershire, UK) coupled with an excitation laser adjusted to a 532 nm wavelength. ICP-OES analysis was carried out using an Ultima 2 tool (Horiba Jobin-Yvon, Longjumenau, France) coupled with a Czerny-Turner-type monochromator with a focal length of 1 m. Commercially available selenium sulfide (Merck, Darmstadt, Germany) served as reference material.

## 3. Results

Together, our investigations have shown that SRB are able to reduce SO_4_^2−^ to H_2_S in the presence of a suitable carbon source, and that this source may indeed also be organic waste material, such as fermented cabbage juice and spoiled milk. Importantly, the H_2_S produced by these bacteria escapes the fermentation culture in millimolar concentrations for up to 310 h and can be collected from the gas phase and reacted directly, for instance, with SeO_2_ to synthesize selenium sulfide. Unlike the fermentation mixture itself, the product formed is highly pure and therefore does not require extensive purification as may be necessary if the reaction was to be performed in the cultivation medium directly.

### 3.1. Fermentation Under Standard Conditions

#### 3.1.1. Production and Quantification of H_2_S

**Figure 2:**
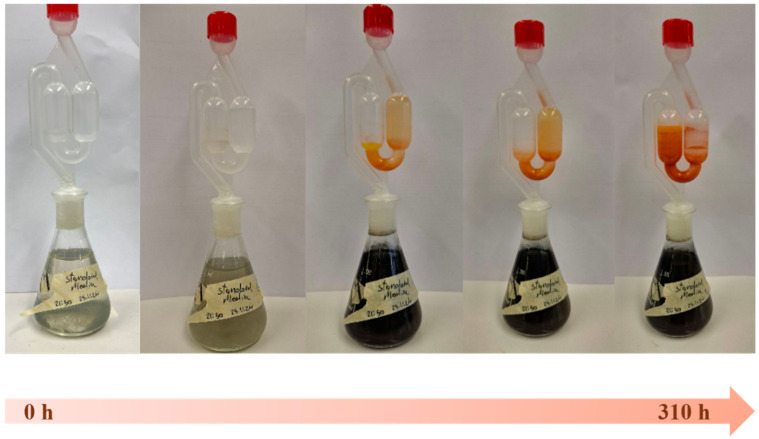
The SRB were cultured in standard media to generate H_2_S which then reacted with SeO_2_ present in the airlock to produce selenium sulfide. A time-dependent increase in the concentration of H_2_S as well as selenium sulfide was observed for up to 310 h.

In the first step, the ability of SRB to convert SO_4_^2−^ to H_2_S in the fermentation equipment was evaluated under standard conditions using the growth medium recommended by the supplier (see DSMZ medium 63: desulfovibrio (postgate) medium). As presented in Figure 2, the fermentation proceeds at 30 °C for almost 13 days, around 310 h, during which time the growth medium transitions in color from yellowish-grey to black.

**Figure 3:**
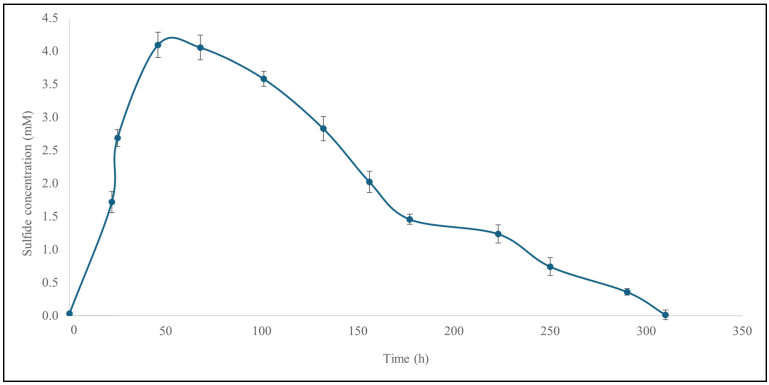
The concentration of H_2_S produced under standard conditions increases rapidly in the gas phase for the first 45 h, reaching 4.1 mM, and then gradually declines over the following 265 h. The experiments were performed in triplicate and on three different occasions (*n* = 9). Results are presented as mean ± SD. See text for further details.

The relevant H_2_S concentration in the gas phase was quantified by the MB assay as described in Section 2.5. A maximum H_2_S concentration of 4.1 mM was observed in the gas phase, gradually increasing from zero over 45 h, and subsequently decreasing over the following 265 h (Figure 3). The concentration of H_2_S in the growth medium itself could not be quantified due to interference of the medium constituents with the MB assay (see Section 4).

#### 3.1.2. Selenium Sulfide Production

The airlock, filled with 15 mL of 100 mM SeO_2_ solution (pH = 2.1), showed the formation of an orange solid starting after around 20 h of incubation. This solid (average weight of 32.7 mg after 310 h, obtained from 75 mL bacterial culture) was collected, washed, and identified as selenium sulfide using a combination of analytical techniques, as explained in Section 2.7. This selenium sulfide produced from biologically generated H_2_S is referred to as “biologically produced selenium sulfide” throughout the manuscript. An overview of the relevant analytical data is provided in Table 1. The yield of selenium sulfide produced in the airlock liquid was calculated to be 15.9% based on the amount of SO_4_^2−^ present in the culture medium and a formal composition of Se_5_S_3_ for the product (see below).

**Figure 4:**
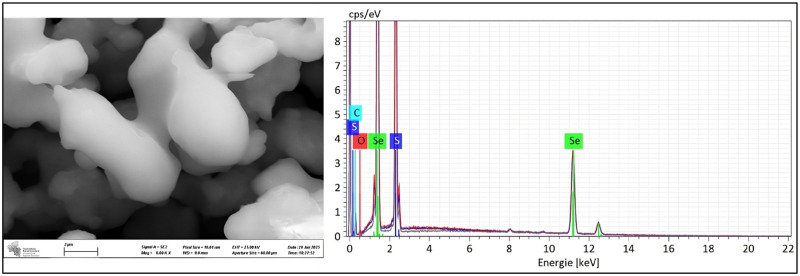
The typical sample of biologically produced selenium sulfide was analyzed using SEM which revealed the presence of agglomerated selenium sulfide particles (**left**). EDX data affirmed the presence of sulfur and selenium in the sample (**right**).

Notably, the elemental selenium-to-sulfur ratio tends to vary slightly from batch to batch, as may be expected for a mixture of 29 different compounds theoretically possible based on eight-membered sulfur–selenium rings with the general formula Se_n_S_8-n_ [16,34]. The ICP-OES analysis revealed mass percentage values corresponding to a molar ratio of 4.8:3.2 (Se:S), aligning with a formula of Se_5_S_3_. EDX analysis provided a ratio of 4.5:3.5 (Se:S), further confirming the predominant average Se_5_S_3_ composition. Essentially, these results suggest an average formal Se to S stoichiometry of 5:3 yet do not allow a firm assignment of exact chemical structures and their respective abundances (Table 1). This ratio is comparably high for selenium, probably due to the excess of SeO_2_ used in the airlock which was deemed necessary to capture the H_2_S gas under these experimental and design conditions. No carbon, hydrogen or nitrogen impurities were detected in the sample according to CHNS analysis, confirming the high purity of the selenium sulfide samples obtained. SEM-EDX was employed to quantify the elemental composition and physical characteristics of the compound and in essence confirmed the presence of selenium and sulfur in the sample (Figure 4). The presence of oxygen was observed by EDX in commercially available selenium sulfide from Merck as well as biologically produced selenium sulfide. Multiple attempts to obtain solid state ^77^Se Nuclear Magnetic Resonance (^77^Se NMR) spectra were unsuccessful, perhaps due to the highly amorphous nature of the sample.

**Figure 5:**
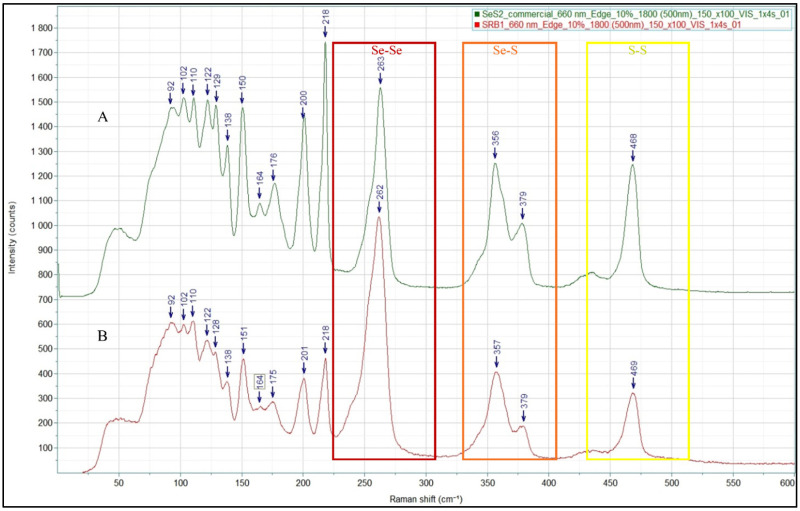
The vibrational modes of biologically produced selenium sulfide (B) provide similar structural fingerprints as that of commercial selenium sulfide (A). Se–S, Se–Se and S–S vibrational stretches are marked in the figure.

The produced selenium sulfide was also analyzed using Raman spectroscopy to investigate the vibrational modes and to establish the structural fingerprints. Commercially available selenium sulfide was analyzed first as reference (Figure 5, spectrum A) followed by biologically generated selenium sulfide (Figure 5, spectrum B). The key Raman vibrational modes of selenium sulfide include Se–Se stretching vibrations occurring around 250 cm^−1^, S–Se stretching vibrations in the range of 300–400 cm^−1^ and S–S stretching vibrations between 400–500 cm^−1^ while peaks with the frequency of lower than 200 cm^−1^ are related to the non-stretching vibration area [35]. A comparative analysis of the Raman spectra of commercial and biologically produced selenium sulfide confirms the presence of all these vibrational modes in our sample, indicating its structural similarity to commercial selenium sulfide (Figure 5).

In addition to the comparison between the Raman spectra of the biosynthesized and commercial selenium sulfide, a spectral comparison between selenium sulfide and the elemental forms of sulfur and selenium was performed. While the Se–S vibrational fingerprint appeared exclusively in the selenium sulfide samples, slight shifts in the Se–Se and S–S vibrational modes were observed compared to the elemental references (Figure S1 in the Supplementary Section).

### 3.2. Variations on the Theme

**Figure 6:**
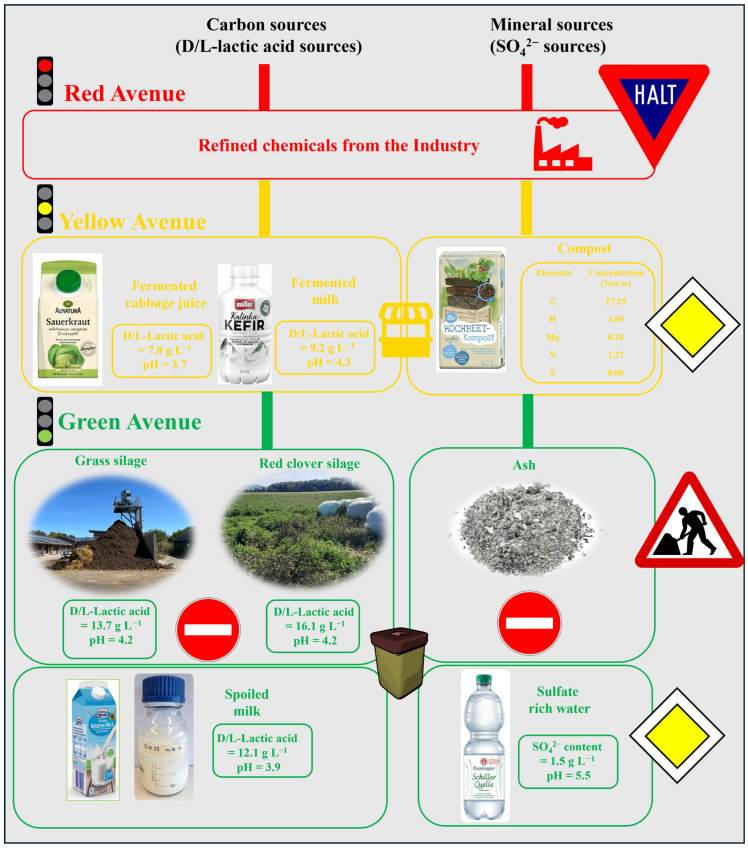
The two main components of standard medium, i.e., DL-lactic acid and minerals were replaced with cheaper and low-grade alternatives for the sake of upcycling organic wastes. Silages (grass and red clover), fermented cabbage juice, fermented milk (kefir) and spoiled milk serve as sources of DL-lactic acid whilst compost, ash and mineral water serve as sources of minerals and especially SO_4_^2−^. The traffic signs indicate which avenues were most promising, closed or are still under construction. Photos are provided by the authors.

In the second step, various components present in the growth medium were either omitted completely or replaced by more conventional materials. These replacements followed a specific traffic-light strategy which went from fine chemicals (red) to less expensive commercial household products (yellow) and eventually waste materials (green). Thus, the replacement strategy in the first step included lactic acid from the drug store, (by-)products of food manufacture and agriculture, and, eventually, organic waste materials. Figure 6 sums up this “replacement” strategy aiming at readily available, ecologically and economically viable substitutes for quality and refined chemicals from industry.

It should be emphasized that the rationale behind this strategy has been to sequentially minimize the number of components and to focus, if possible, on waste materials available in bulk quantities. Thus, not every theoretically possible combination of materials was tried, since several combinations have been deemed worth investigating further, whereas others were not considered as such. Furthermore, this section of the study also did not yet aim at optimization of the various processes. The selection of waste materials was primarily based on the presence of key ingredients required for the bacterial growth. Fermented cabbage juice, kefir, red clover silage, grass silage and spoiled milk are rich in DL-lactic acid whilst compost, ash and mineral water serve as source of essential minerals and SO_4_^2−^.

Eventually, it was possible to minimize the number and amount of fine chemicals and conduct the fermentation successfully with either a combination of commercial fermented cabbage juice and compost (see Section 3.2.1), or with spoiled milk and mineral water (see Section 3.2.3).

#### 3.2.1. The Sauerkraut Connection

The first replacement avenue takes advantage of the fact that SRB require DL-lactic acid as a carbon source. The juice of fermented cabbage, naturally containing between approximately 0.61 g L^−1^ and 11.60 g L^−1^ DL-lactic acid, mostly thanks to *Lactobacillus* bacteria converting lactose to DL-lactic acid, could be used as an alternative carbon source [36]. Although rich in DL-lactic acid and minerals, fermented cabbage juice does not contain sulfate to support H_2_S production. The need for an additional sulfate source could be met by adding compost, which is rich in carbon, nitrogen, phosphorous and sulfur and thus seems to be sufficient to supply the missing components that are not naturally present in fermented cabbage (Sauerkraut) juice, such as SO_4_^2−^ ions. The composition of compost was analyzed using CHNS analysis alongside the data provided by the manufacturer (Figure 6).

**Figure 7:**
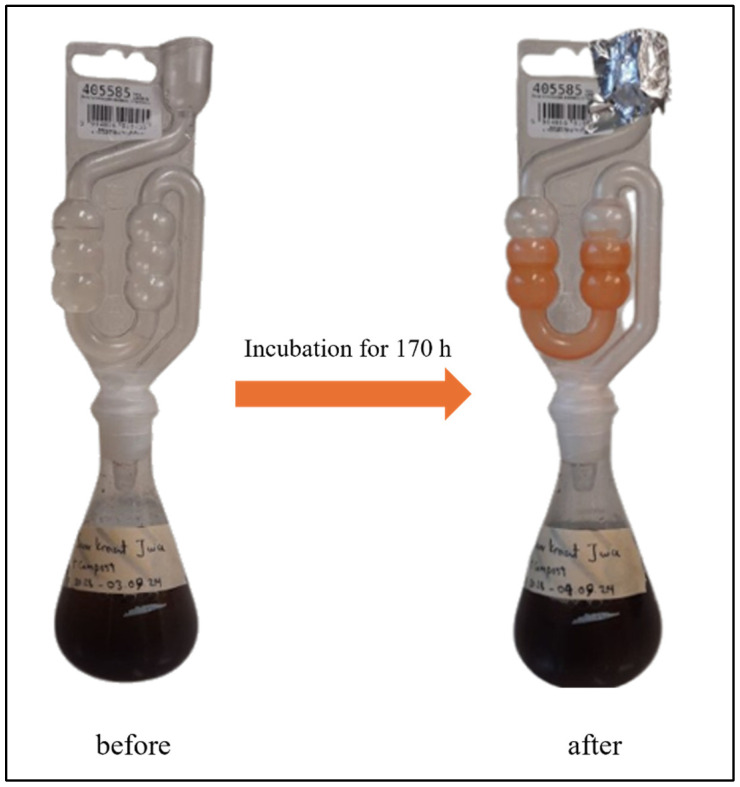
A mixture of fermented cabbage juice and compost was utilized as growth medium to culture SRB. The pH was adjusted to 7.0 using a few drops of ammonium hydroxide (NH_4_OH). The resultant H_2_S produced by the bacteria can be exploited to produce selenium sulfide.

The “minimal Kraut medium” (comprising of Sauerkraut juice and compost) was able to support the reduction of SO_4_^2−^ to H_2_S by the SRB culture as shown in Figure 7. Compared to the conventional growth medium, the yields of H_2_S in the gas phase and thus selenium sulfide seemed to be lower (an average of 5 mg selenium sulfide was collected from 75 mL culture after 170 h), yet this is hardly surprising as the fermentation was not fully optimized during this initial feasibility study. The amount of selenium sulfide obtained was insufficient to perform the complete physical characterization analysis.

#### 3.2.2. The Silage Saga

**Figure 8:**
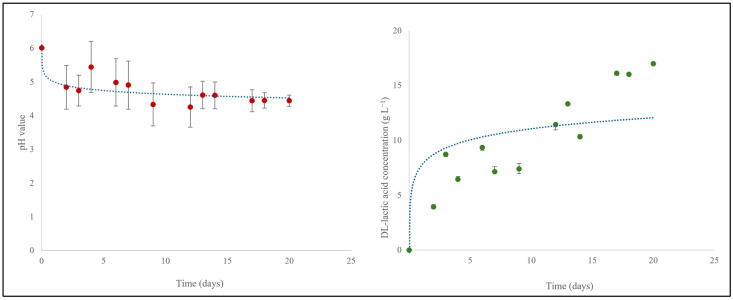
Red clover silage was produced in the laboratory from the clover collected at Saarland University and showed a time-dependent decrease in pH from 6.0 to 4.2 (**left**) and increase in the DL-lactic acid concentration from 0 to 16.1 g L^−1^ (**right**) over a period of 20 days. The experiments were performed in triplicate and on three different occasions (*n* = 9). Results are presented as mean ± SD. See the text for further details.

Fermented cabbage–and its juice–is not necessarily a waste material, and thus this avenue was not investigated further. A less valuable alternative was therefore considered and found in the juice produced during the silage process which indeed mirrors the Sauerkraut fermentation with Lactic Acid Bacteria (LAB) yet yields a worthless liquid. Notably, the starting materials for silage, such as red clover, are not heat sterilized or salted, thus the silage juice is not only rich in DL-lactic acid (16.1 g L^−1^) and fairly acidic (pH = 4.2); it also contains a wide range and variety of other compounds and components and therefore is often considered as an outright nuisance by farmers (Figure 8). Regardless if “real life” grass silage from the Bannsteinhof farm, or homemade red clover silage was used in place of cabbage juice, the SRB did no longer flourish or produce selenium sulfide. This is somewhat disappointing since the homemade silage did contain up to 16.1 g L^−1^ DL-lactic acid and with a pH of 4.2 in these important aspects was not that dissimilar to the commercial cabbage juice employed successfully in Section 3.2.1.

#### 3.2.3. The Milky Way

In addition to fermented cabbage juice, DL-lactic acid is also found in fermented milk products such as yogurt and kefir, both of which contain up to 12 g L^−1^ and 10 g L^−1^ DL-lactic acid, respectively [37,38]. In these food products, bacteria such as *Lactobacillus* species convert lactose to DL-lactic acid. DL-lactic acid also often forms when milk expires and simply “goes off” and the resulting spoiled milk may also contain up to 10% DL-lactic acid [37]. In fact, the bacteria responsible for this conversion of lactose to DL-lactic acid are omnipresent, infecting many natural products, from red clover to milk. Moreover, the non-growing starter cultures also pose a severe challenge to the food industry due to their prolonged metabolic activity during storage leading to post-production acidification. In stored yogurts, for instance, slow but continuous lactic acid generation causes not only shorter shelf life but also considerable alterations in flavor as well as acidity [39,40,41,42].

**Figure 9:**
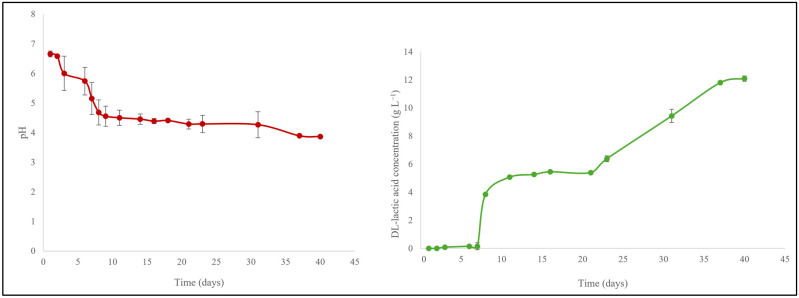
A time-dependent decrease in pH from 6.7 to 3.9 (**left**) and an increase in the DL-lactic acid concentration from 0 to 12.1 g L^−1^ (**right**) was observed for milk which was allowed to rot at room temperature for 40 days. The experiments were performed in triplicate and on three different occasions (*n* = 9). Results are presented as mean ± SD. See text for further details.

Commercial kefir containing 9.2 g L^−1^ D/L-lactic acid (pH 4.3) was therefore investigated and turned out to be a rather good source of D/L-lactic acid for SRB fermentation in combination with Na_2_SO_4_. In order to replace commercial kefir with spoiled milk, 1.5% homogenized low-fat milk was obtained from the local Netto supermarket and simply allowed to “rot” at room temperature for up to 40 days. During this time, the concentration of DL-lactic acid in the sample increased from 0 to 12.1 g L^−1^, whilst the pH value continuously dropped from pH 6.7 to pH 3.9 as shown in Figure 9. After 40 days, this lactose-to-DL-lactic acid “fermentation” was complete as indicated by a more or less steady pH value of 3.9 and a DL-lactic acid concentration of 12.1 g L^−1^.

**Figure 10:**
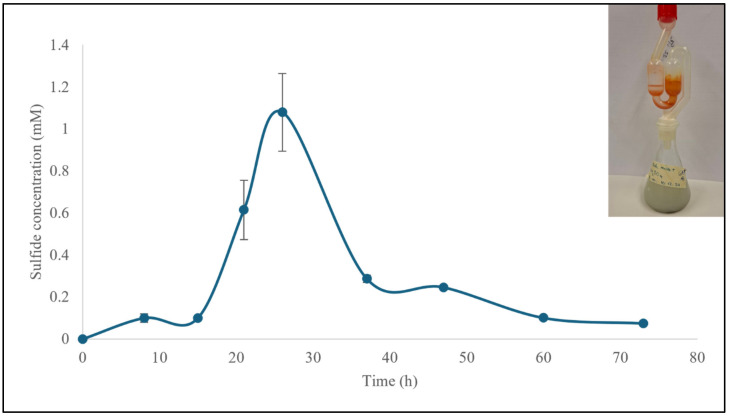
SRB were able to produce H_2_S when a mixture of spoiled milk and Na_2_SO_4_ was used as culture medium. H_2_S was produced over 60 h in the headspace, reaching a maximum concentration of just above 1 mM after 25 h. The experiments were performed in triplicate and on three different occasions (*n* = 9). Results are presented as mean ± SD. See text for further details.

Spoiled milk can be used in combination with a SO_4_^2−^ source, in this case Na_2_SO_4_, to provide essential nutrients for the growth of SRB and the production of H_2_S. Inoculation of this culture with SRB resulted in the generation of up to 1 mM H_2_S in the headspace (Figure 10). The H_2_S was further reacted with SeO_2_ to provide selenium sulfide which was subsequently characterized using the above-mentioned techniques. The results are presented in Supplementary Section Table S3 and Figures S2 and S3.

**Figure 11:**
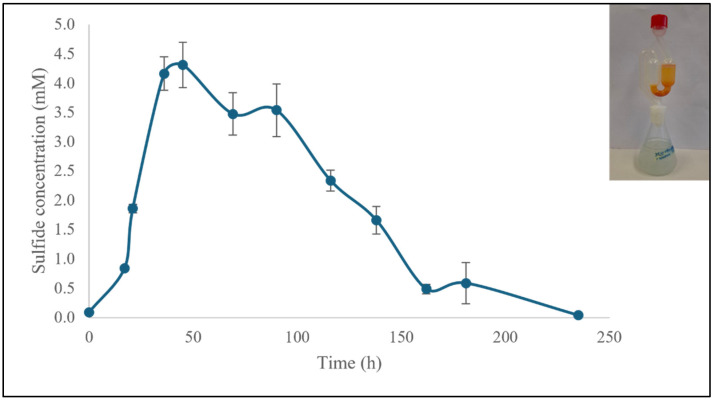
SRB were able to produce H_2_S when a mixture of spoiled milk and mineral water was used as culture medium. A time-dependent increase in the concentration of H_2_S was observed in the headspace, reaching more than 4.3 mM, followed by a gradual decrease in the subsequent hours. The experiments were performed in triplicate and on three different occasions (*n* = 9). Results are presented as mean ± SD. See text for further details.

Alternatively, SO_4_^2−^ can be replaced with sulfate found in natural sources such as mineral waters. One of these natural mineral water sources is the SO_4_^2−^ rich spa water originating from the natural gypsum-rich underground sedimentary rock formations around Ensingen in Baden-Wuerttemberg (Germany, 48°58′ N, 8°57′ E) and reportedly containing 1.5 g L^−1^ of SO_4_^2−^ [43]. Following the successful inoculation of SRB in a 1:2 mixture of spoiled milk and mineral water neutralized by a few drops of NH_4_OH, H_2_S production in the headspace after 45 h exceeded an astonishing 4.3 mM before gradually declining over the next 190 h (Figure 11). Thus, the “redneck” fermentation with spoiled milk and Ensinger mineral water was as efficient as the one following the standard protocol and employing various expensive fine chemicals (see Figure 11).

After 235 h, an average of 48.3 mg of selenium sulfide was obtained. The selenium sulfide was then thoroughly washed (see Section 2.6), dried and subsequently analyzed. CHNS analysis, ICP-OES, and EDX confirmed the presence of selenium and sulfur in the sample. The elemental composition is more similar to the commercial selenium sulfide compared to the standard medium, with the Se to S ratio in ICP-OES corresponding to 3:5 and in EDX corresponding to 2.8:5.2, resulting in the overall stoichiometric ratio of approximately 1:2 (Supplementary Section, Table S4, Figure S4). CHNS analysis also affirmed the absence of any carbon, hydrogen and nitrogen in the sample. The Raman spectroscopy confirmed similar structural fingerprints of selenium sulfide as those of standard selenium sulfide from Merck (Supplementary Section, Figure S5).

## 4. Discussion

In essence, the successful production of selenium sulfide from SeO_2_ and H_2_S produced via anaerobic SRB fermentation of a mixture of spoiled milk and Ensinger mineral water confirms the feasibility of a “redneck” chemistry using “dirty” waste materials as one of the relevant components in place of commercially available fine chemicals. Although the processes underlying these fermentations still could be refined and optimized in order to achieve even higher yields and thus fulfil economic prospects, this feasibility study already demonstrates and underlines a few important points.

First and foremost, microbial fermentation seems to be a valuable avenue to generate H_2_S under mild conditions, at room temperature and from readily available organic (by-) products such as spoiled milk and natural spa waters as well as a combination of fermented cabbage juice and compost. This differs from ccommercial H_2_S which is either recovered from gas mixtures or produced chemically. Both of these means are energy intensive as well as environmentally harmful. Compared to our previous studies utilizing H_2_S-rich natural waters, H_2_S produced by fermentation is more accessible and also less tedious to collect and to process. The concentrations of 4.1–4.3 mM in the headspace of the airlock, for instance, compare favorably with the mineral waters used in our previous study, which contained only up to 2.4 mM H_2_S. The escape of H_2_S gas from such sulfide-rich springs reduces its available sulfur content. Therefore, sulfate-rich mineral water such as Ensinger Schiller Quelle serves as a better and more reliable sulfur source with a SO_4_^2−^ concentration of around 15.6 mM, providing more than six times the sulfur content of the H_2_S-rich Bad Nenndorf spring water used previously and without the risk of SO_4_^2−^ escaping in the gas phase.

The collection of the selenium sulfide produced from the water from Bad Nenndorf was also tedious as it required extensive filtration. Using SRB, H_2_S can be produced from SO_4_^2−^ rather easily in higher concentrations and also, literally, escapes the dirty fermentation mixture and can be captured continuously in the airlock. This strategy avoids the need for subsequent filtration or purification steps and also enables an easy harvest of considerable amounts of product.

Indeed, besides simple low-energy fermentation, purification is the second most relevant cornerstone of this fermentation process. There are reports in the literature describing the production of selenium sulfide using yeast cultured in the presence of selenite (SeO_3_^2−^) and sulfite (SO_3_^2−^) [44]. Considering the simplicity of selenium sulfide production in such a culture, this process at first seems to be highly attractive. Yet, despite the fact that some selenium sulfide may indeed have formed under these conditions, purification of the material is tedious, cumbersome and, in our opinion and experience, neither economical nor especially ecological. It is also not possible to determine or use H_2_S concentrations in such “dirty” media directly, employing, for instance, the MB assay, as mentioned above. This also applies to the reaction of H_2_S with SeO_2_ in the medium, as a product formed in the medium is difficult to purify.

Thus, separating the fermentation process on the one side from the product formation and purification process on the other has considerable attraction. In this context, H_2_S is therefore of special interest, as it escapes the culture in a more or less pure form together with other gases such as CO_2_ and subsequently can be captured and used as a starting material for further products, in our case selenium sulfide. Other H_2_S-based compounds, such as thiazolidine-2-one derivatives, are, of course also in the pipeline/airlock as attractive molecules using a similar setup for their synthesis [45]. In addition to organic and inorganic compounds, one may also employ H_2_S directly, for instance in fuel cells [46].

The setup itself has been designed as a simple, cost-effective and robust system, and indeed, the use of the airlock as a combination of traditional airlock for anaerobic fermentation and simultaneously a reaction vessel has proved an elegant solution to the anaerobic SRB fermentation, at least on the laboratory scale. Upscaling and refining this process is, of course, possible, and this may indeed include the collection of H_2_S and its storage or transport to a separate reaction chamber. It is important to mention that the volume of headspace may also affect the production of H_2_S and this could be fine-tuned together with other relevant variables, such a volume of media and temperature [47]. This is an issue of technical (bio-) chemistry that may be addressed in subsequent studies considering the optimization of this process.

Optimization itself may include a finer adjustment of individual components or eventually the use of mutant strains of SRB able to produce H_2_S in higher yields. The choice of fermented cabbage juice as a replacement for commercial DL-lactic acid has been a good starting point, since this juice is more sustainable and thus considerably cheaper than industrially produced DL-lactic acid. Cabbage waste is a significant issue in the agricultural sector. In the cabbage market, the global production of cabbage was estimated at 70 million tons in 2018 and is projected to reach 88 million tons in 2025 with an average wastage rate of 14%, according to data from Gauteng, South Africa [48,49,50]. In theory, this accounts for approximately 16 million tons of discarded cabbage for 2025, which could be recovered and repurposed as a sustainable carbon source, along with expired food-grade cabbage juice [50,51,52]. Of course, fermented cabbage–and its juice–is not necessarily a waste material. In Germany, large crowds of Krauts simply love their Sauerkraut and consume its juice like table water *(personal communication by the corresponding author).*

Although initial attempts to move on from cabbage juice and to utilize silage or silage juices have failed due to their apparent toxicity, this avenue is indeed still very promising and may need to be revisited again in the hope that some SRB or other lactate-consuming bacteria may be more resistant and thus able to thrive in the presence of this liquid. Considering that silage juice is rich in many nutrients, it may also be possible, for instance, to pretreat it before using it in SRB culture or to switch from red clover to maize or another green waste material just as it was possible to eventually switch from cabbage juice to spoiled milk. In any case, the silage saga must go on as silage juice is a major side-product of agricultural fermentation and so far more of a nuisance to farmers and the environment than a potential mine for sustainable processes—and therefore fulfils many of the conditions for turning this inherent waste into considerable value—easily and on a very large scale indeed.

Then again, milk is among the most discarded food items today. In the UK, for instance, it comes second on the list of most wasted food items right after wasted bread and before wasted bananas. Each year, around 500,000 tons of milk literally go down the drain in the UK alone, and this milk may be used in fermentation, together with the tons of dairy products, yogurt, kefir, etc., also dumped each year [53,54,55]. This concept becomes particularly appealing as the amount of selenium sulfide produced using spoiled milk and the spa water from Ensinger Schiller Quelle exceeds the amount obtained with standard media. Unlike fermented cabbage juice, which is not universally considered a waste material, spoiled milk is clearly a waste product. For these reasons, spoiled milk and mineral water from rocks rich in gypsum emerge as the most promising alternatives to the standard medium in this experiment.

Ideally, a sustainable process not only requires “worthless” or even “nuisance” educts—it should also minimize its own waste. As for the waste produced by this microbial fermentation, neither the compost, the spoiled milk nor the SRB pose any environmental risk and thus may be discarded after use, for instance for further downstream fermentation in biogas production. SRB are harmless soil bacteria found, for instance, in soils, freshwater and saltwater ecosystems, sediments, mud and even the rhizosphere of plants and, in any case, are sensitive to dioxygen, and thus unlikely to pose any danger to plants, animals or humans [56,57]. A mixed culture of SRB can, therefore, be exploited in the future for further fine-tuning of this robust system of H_2_S production. Since the amount of Fe^2+^ ions also impacts the production of H_2_S, one could sprinkle a pinch of iron and steel slag or red mud on the growth media to further enhance the efficiency of the set up, and could also consider gypsum waste from building sites in place of mineral waters [47,58].

Eventually, the notion of using cheap organic by-products or waste as carbon sources to fuel the microbial fermentation to produce valuable (raw) chemicals may be expanded further. Considering that such fermentations are indeed “robust” or, as one may call it, “dirty”, conventional extractions and purifications may be difficult and expensive, thus focussing on “phase separations”, for instance, by involving gasses, may be the most economical way forward. Here, H_2_S stands out, also thanks to its low solubility in aqueous media (117 mmol L^−1^ at 20 °C), yet it is not alone, and other biologically generated gasses may also be produced by waste fermentation. In addition to the more usual suspects, such as methane (CH_4_) and hydrogen (H_2_), they also include chemically interesting gases such as ammonia (NH_3_) from nitrogen-rich waste materials, including urine.

## 5. Conclusions

In order to make chemical and pharmaceutical processes sustainable, one could consider a number of strategies, from recycling to replacement. Turning organic waste into value is one of these promising avenues since it utilizes readily available yet hitherto worthless or even nuisance materials. Although there is no free lunch, there are plenty of by-products and leftovers on the table which can be used, from cabbage juice and silage on one the side to spoiled milk, natural mineral water and compost on the other. These materials, together with inorganic wastes, such as gypsum, are omnipresent in modern society, they can be found in considerable quantities and, thanks to a continued supply, are unlikely to run out in the near future.

The real challenge is to find or produce valuable materials from this waste without excessive processing or purification which eventually may negate the economical or ecological gains. Indeed, stimulating chemistry in a waste environment is far from trivial, and purification may be like looking for the literal needle in the haystack—or gold in a French soccer star’s manure—a cumbersome and unpleasant experience of little economical value [59].

In this context, fermentation by robust (soil) bacteria may resolve some of these issues and involving a gas such as H_2_S or NH_3_ certainly provides an elegant solution to many of these problems. In the future, these processes need to be studied, optimized and upscaled further in order to gain ecological and economic value. Based on our initial studies presented here, this seems to be possible. To put is poetically, it is not a sham—the right design may really turn poo into shampoo!

## Figures and Tables

**Table 1:** Characterization of the selenium sulfide produced using standard medium.

Characterization Method	Biologically Produced Selenium Sulfide (wt%)	Commercial Selenium Sulfide (wt%)
CHNS	S: 35.02	S: 47.98
ICP-OES	S: 21.50	S: 43.21
	Se: 78.50	Se: 56.79
EDX	S: 24.05	S: 45.02
	Se: 75.95	Se: 54.98

## Data Availability

The original contributions presented in this study are included in the article and Supplementary Materials. Further inquiries can be directed to the corresponding authors.

## Supplementary Materials

The following supporting information can be downloaded at https://www.mdpi.com/article/10.3390/ma18122784/s1, Table S1: The gradual elimination of ingredients of standard medium to determine the most essential ingredients required for the growth of *D. desulfuricans*; Table S2: Substitution of ingredients of standard medium with more common/waste ingredients; Table S3: The elemental composition of selenium sulfide harvested from the SRB cultured in spoiled milk and Na_2_SO_4_. The Se:S ratio slightly differs from that obtained from the standard medium, as confirmed by CHNS, ICP-OES and EDX analysis; Table S4: The elemental composition of selenium sulfide harvested from the SRB cultured in a 1:2 mixture of spoiled milk and mineral water analyzed by CHNS, ICP-OES and EDX analysis; Figure S1: Raman spectra of elemental selenium, elemental sulfur, commercial selenium sulfide and biosynthesized selenium sulfide show that the Se–S vibrational band appears only in the selenium sulfide samples, while slight shifts in the Se–Se and S–S modes are observed compared to the elemental references; Figure S2: The spoiled milk and Na_2_SO_4_ mediated selenium sulfide powder was analyzed using SEM which revealed the presence of agglomerated selenium sulfide particles (left). The presence of sulfur and selenium at 34.56 wt% and 65.44 wt% was affirmed by EDX (right); Figure S3: The structural fingerprints of selenium sulfide produced using a mixture of spoiled milk and Na_2_SO_4_. The sample selenium sulfide (A) and commercial selenium sulfide (B). Similar vibrational modes could be observed between biologically produced selenium sulfide and commercial selenium sulfide; Figure S4: Selenium sulfide produced using the spoiled milk and mineral water was analyzed using SEM (left). EDX analysis confirmed the presence of sulfur and selenium at 39.29 wt% and 60.71 wt%, respectively (right); Figure S5: The comparison of the structural fingerprints of selenium sulfide produced using a mixture of spoiled milk and mineral water (A) and commercial selenium sulfide (B) as well as the standard medium mediated selenium sulfide (C).
