# Supplementary material for: Valorizing Organic Waste: Selenium Sulfide Production Mediated by Sulfate-Reducing Bacteria
Source: Materials (Basel). 2025 Jun 13;18(12):2784. doi: 10.3390/ma18122784 (PMC12195272; doi:10.3390/ma18122784)
Supplement: Supplementary file 1 [file materials-18-02784-s001.zip › materials-3598033-supplementary.pdf]

materials

MDPI

Article

Valorizing Organic Waste: Selenium Sulfide Production Mediated by Sulfate-Reducing Bacteria

Shahrzad Safinazlou [1], Ahmad Yaman Abdin [1], Eduard Tiganescu [1], Rainer Lilischkis [2], Karl-Herbert Schäfer [3], Claudia Fink-Straube [4], Muhammad Jawad Nasim [1,*] and Claus Jacob [1,*]

1 Division of Bioorganic Chemistry, School of Pharmacy, Saarland University, 66123 Saarbruecken, Germany, shsa00006@uni-saarland.de (S.S.); yaman.abdin@uni-saarland.de (A.Y.A.); s9edtiga@stud.uni-saarland.de (E.T.);

2 Working Group Informatic and Microsystems Technology Department, University of Applied Sciences Kaiserslautern, Amerikastrasse 1, 66482 Kaiserslautern, Germany, rainer.lilischkis@hs-kl.de (R.L.);

3 Working Group Enteric Nervous Systems (AGENS), University of Applied Sciences Kaiserslautern, Amerikastrasse 1, 66482 Kaiserslautern, Germany, karlherbert.schaefer@hs-kl.de (K.H.S.)

4 INM–Leibniz Institute for New Materials, 66123 Saarbrücken, Germany, claudia.fink-straube@leibniz-inm.de (C.F.S.)

* Correspondence: jawad.nasim@uni-saarland.de (M.J.N.); c.jacob@mx.uni-saarland.de (C.J.); Tel.: +49-681-302-57335 (M.J.N.); +49-681-302-3129 (C.J.)

 https://doi.org/10.3390/ma18122784

Table S1. The gradual elimination of ingredients of standard medium to determine the most essential ingredients required for the growth of *D. desulfuricans*

**K_2HPO_4**	**NH_4Cl**	**Na_2SO_4**	**$CaCl_2$.• $2H_2O$**	**$MgSO_4$• $7H_2O$**	**SodiumDL-lactate**	**Yeast extract**	**sodium resazurin**	**$FeSO_4$• $7H_2O$**	**Sodium thioglycolate**	**Ascorbic acid**	**Bacterial growth**
				✓	✓						No growth
		✓			✓						No growth
✓	✓	✓	✓	✓	✓	✓	✓	✓		✓	Optimal growth
✓	✓	✓	✓	✓	Substitution with lactic acid	✓	✓	✓	✓	✓	Optimal growth
✓	✓	✓	✓	✓		✓	✓	✓	✓	✓	Suboptimal growth
✓	✓	✓	✓	✓	✓		✓	✓	✓	✓	Optimal growth
✓	✓	✓	✓	✓	✓	✓		✓	✓	✓	Optimal growth
✓	✓	✓	✓	✓	✓	✓	✓		✓	✓	Optimal growth
✓	✓	✓	✓		✓	✓	✓				Optimal growth
✓	✓		✓	✓	✓	✓	✓				Optimal growth
✓		✓	✓	✓	✓	✓	✓				Optimal growth
✓	✓	✓		✓	✓	✓	✓				Optimal growth
✓		✓		✓	✓	✓	✓				Suboptimal growth
	✓	✓	✓	✓	✓	✓	✓				Optimal growth
✓	✓		✓		✓	✓	✓				Optimal growth
		✓		✓	✓	✓	✓				Optimal growth
		✓		✓	✓	✓					Optimal growth
		✓			✓	✓					Optimal growth

				✓	✓	✓					Optimal growth
✓	✓	✓	✓	✓			✓				Suboptimal growth
					✓	✓					Optimal growth

Table S2. Substitution of ingredients of standard medium with more common/waste ingredients.

**Organic ingredients**	**Inorganic ingredients**	**Bacterial growth**
Silage (autoclaved)	$MgSO_4•7H_2O$<br>K_2HPO_4<br>$CaCl_2•2H_2O$<br>$FeSO_4•7H_2O$	• No growth
Silage (not autoclaved)	$MgSO_4$ $•7H_2O$<br>K_2HPO_4<br>$CaCl_2•2H_2O$<br>$FeSO_4•7H_2O$	• No growth
Silage (autoclaved)	$MgSO_4•7H_2O$	• No growth
Red clover silage<br>Compost	$MgSO_4•7H_2O$	• No growth
Red clover silage	$MgSO_4.7H_2O$	• No growth
Red clover silage	$MgSO_4•7H_2O$	• No growth
Fermented cabbage juice	$MgSO_4•7H_2O$<br>K_2HPO_4<br>$CaCl_2•2H_2O$<br>$FeSO_4•7H_2O$	• Optimal
Fermented cabbage juice	$MgSO_4•7H_2O$	• No growth

Compost		
Fermented cabbage juice	Na_2SO_4	• No growth
Fermented cabbage juice	$MgSO_4{\bullet}7H_2O$	• Suboptimal growth
Fermented cabbage juice	$CaSO_4$	• Suboptimal growth
Fermented cabbage juice<br>Ash		• No growth
Fermented cabbage juice<br>Compost		• Suboptimal growth
Supernatant of kefir<br>Compost	$MgSO_4{\bullet}7H_2O$<br>NH_4OH	• No growth
Supernatant of kefir	$MgSO_4{\bullet}7H_2O$<br>Ash	• No growth
Supernatant of kefir	$MgSO_4{\bullet}7H_2O$<br>NH_4OH	• Suboptimal growth
Supernatant of kefir<br>Compost		• Suboptimal growth
Spoiled milk<br>Compost	$MgSO_4{\bullet}7H_2O$	• No growth
Spoiled milk<br>Ash		• No growth
Spoiled milk<br>Compost		• Suboptimal growth
Spoiled milk	Na_2SO_4	• Optimal
Spoiled milk	Mineral water	• Optimal

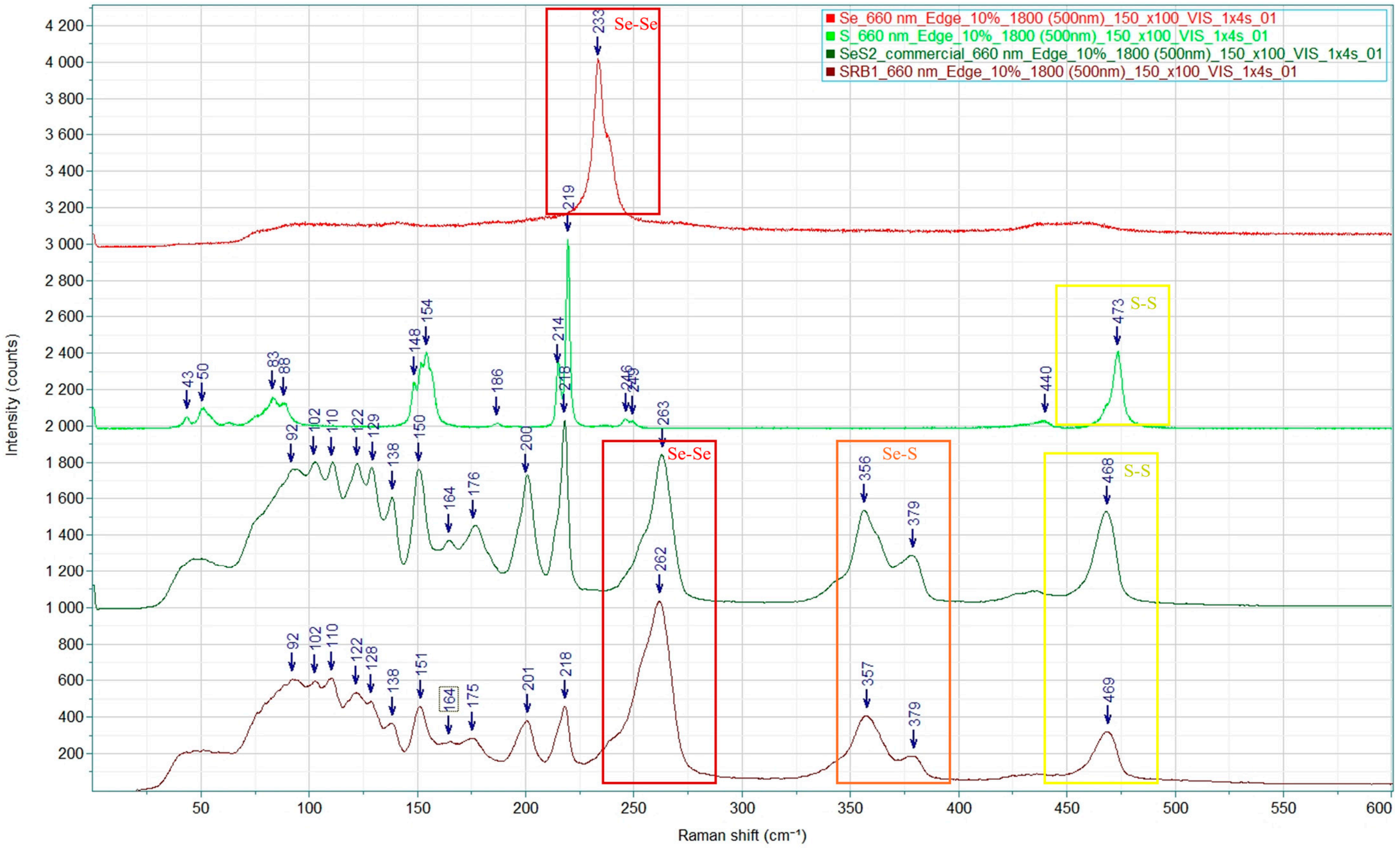

Figure S1. Raman spectra of elemental selenium, elemental sulfur, commercial selenium sulfide, and biosynthesized selenium sulfide show that the Se–S vibrational band appears only in the selenium sulfide samples, while slight shifts in the Se–Se and S–S modes are observed compared to the elemental references.

Table S3. The elemental composition of selenium sulfide harvested from the SRB cultured in spoiled milk and Na_2SO_4. The Se:S ratio slightly differs from thatobtained from the standard medium, as confirmed by CHNS, ICP-OES, and EDX analysis

Characterization method	Selenium sulfide Sample (wt%)	Commercial selenium sulfide (wt%)
CHNS	S: 55.13	S: 47.98
ICP-OES	S: 32.07	S: 43.21
	Se: 67.93	Se: 56.79
EDX	S: 34.56	S: 45.02
	Se: 65.44	Se: 54.98

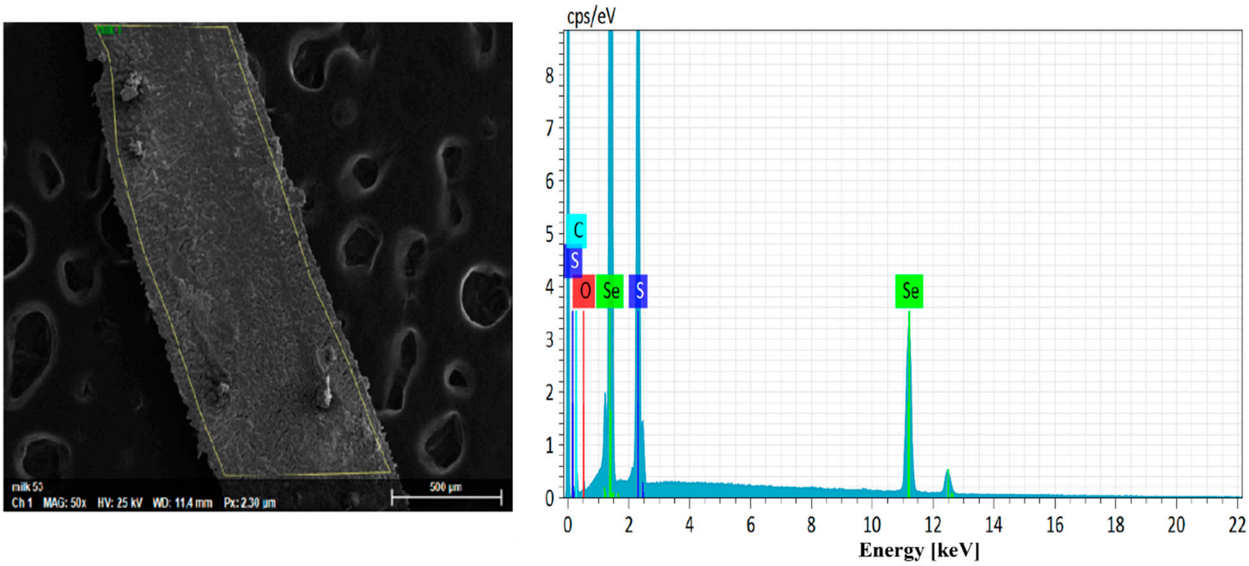

Figure S2. The spoiled milk and Na_2SO_4 mediated selenium sulfide powder was analyzed using SEM which revealed the presence of agglomerated selenium sulfide particles (left). The presence of sulfur and selenium at 34.56 wt%and 65.44 wt% was affirmed by EDX (right).

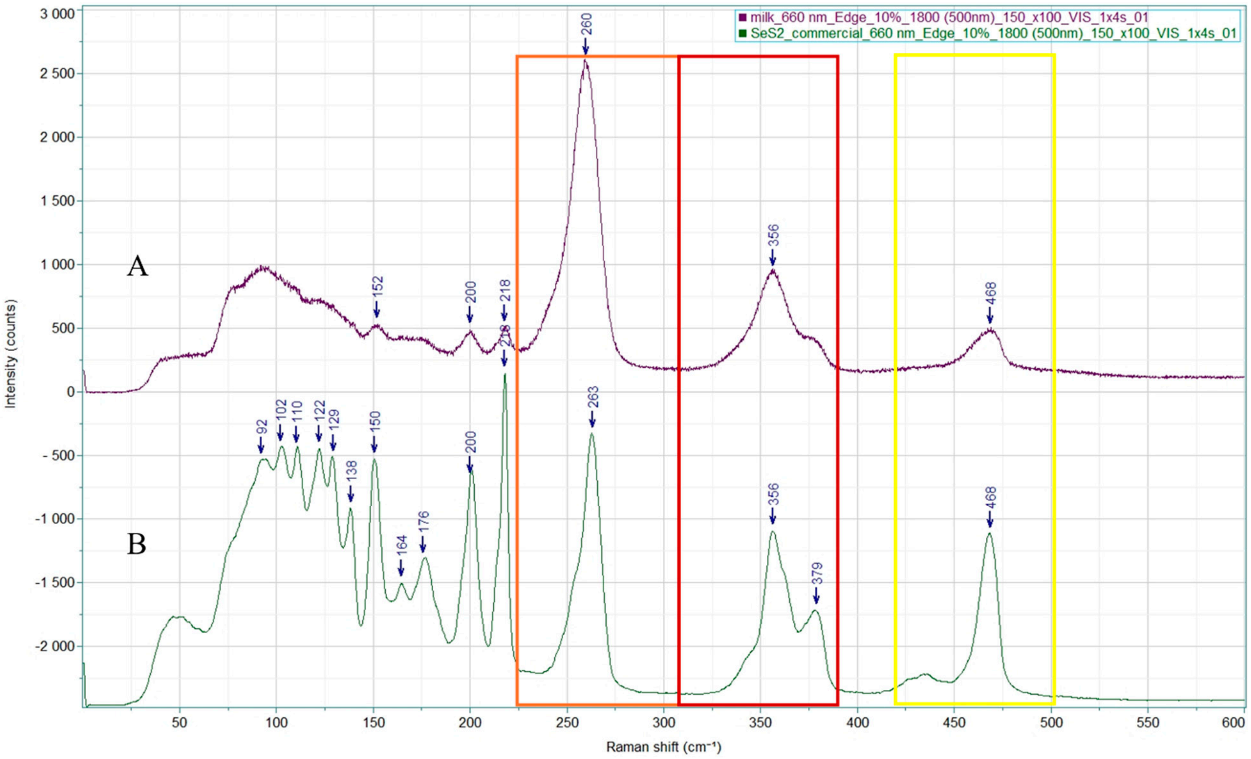

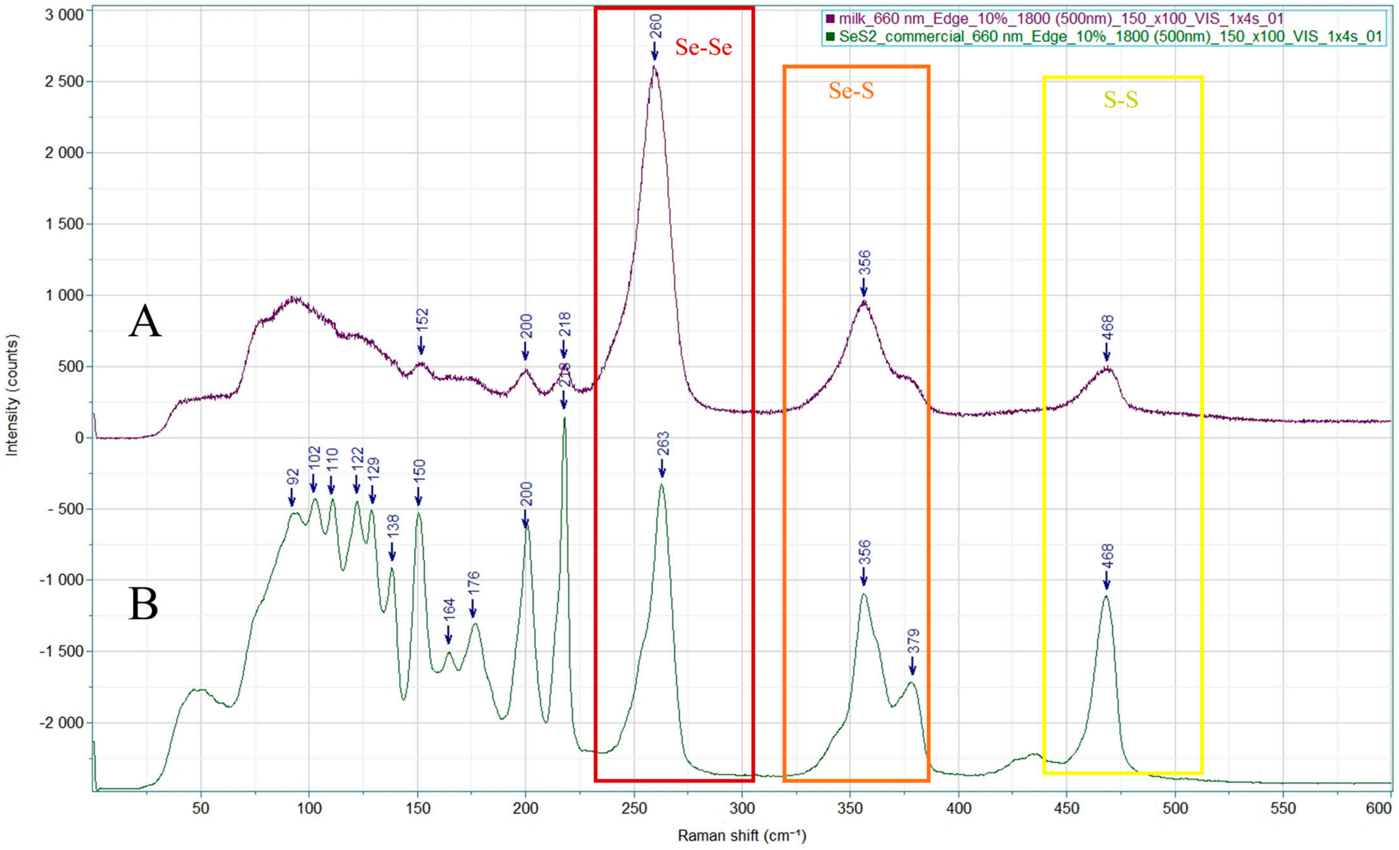

Figure S3. The structural fingerprints of selenium sulfide produced using a mixture of spoiled milk and Na_2SO_4. The sample selenium sulfide (A) and commercial selenium sulfide (B). Similar vibrational modes could be observed between biologically produced selenium sulfide and commercial selenium sulfide.

Table S4. The elemental composition of selenium sulfide harvested from the SRB cultured in a 1:2 mixture of spoiled milk and mineral water analyzed by CHNS, ICP-OES, and EDX analysis.

Characterization method	Selenium sulfide sample (wt%)	Commercial selenium sulfide (wt%)
CHNS	S: 44.34	S: 47.15
ICP-OES	S: 40.75	S: 42.20
	Se: 59.25	Se: 57.80
EDX	S: 39.29	S: 42.23
	Se: 60.71	Se: 57.76

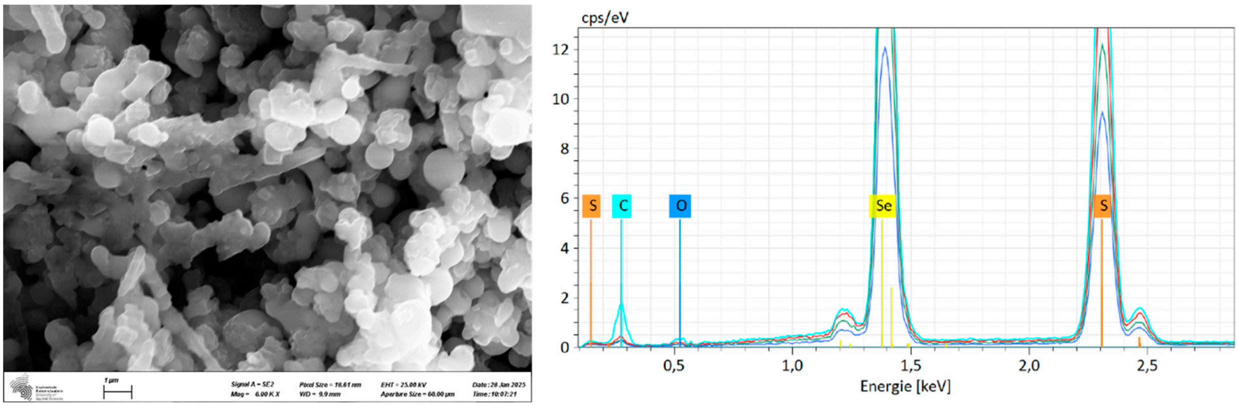

Figure S4. Selenium sulfide produced using the spoiled milk and mineral water was analyzed using SEM (left).EDX analysis confirmed the presence of sulfur and selenium at 39.29 wt% and 60.71 wt%, respectively (right).

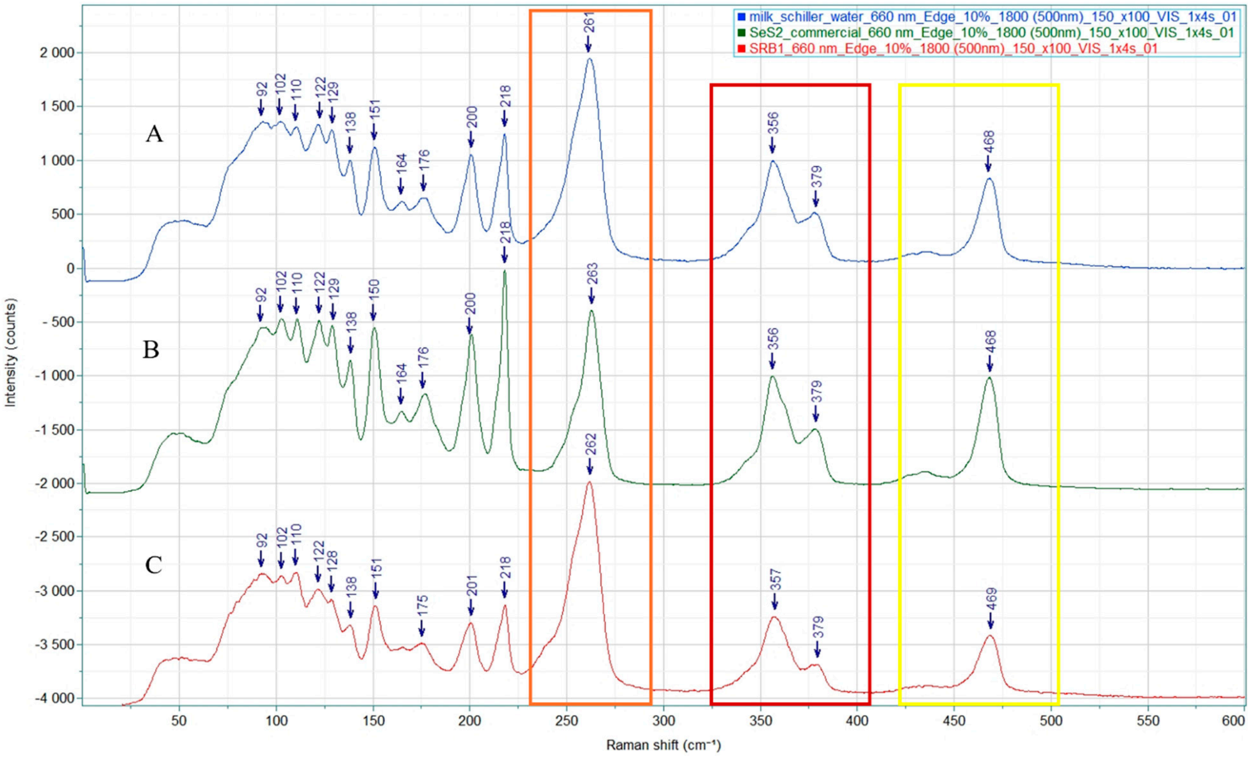

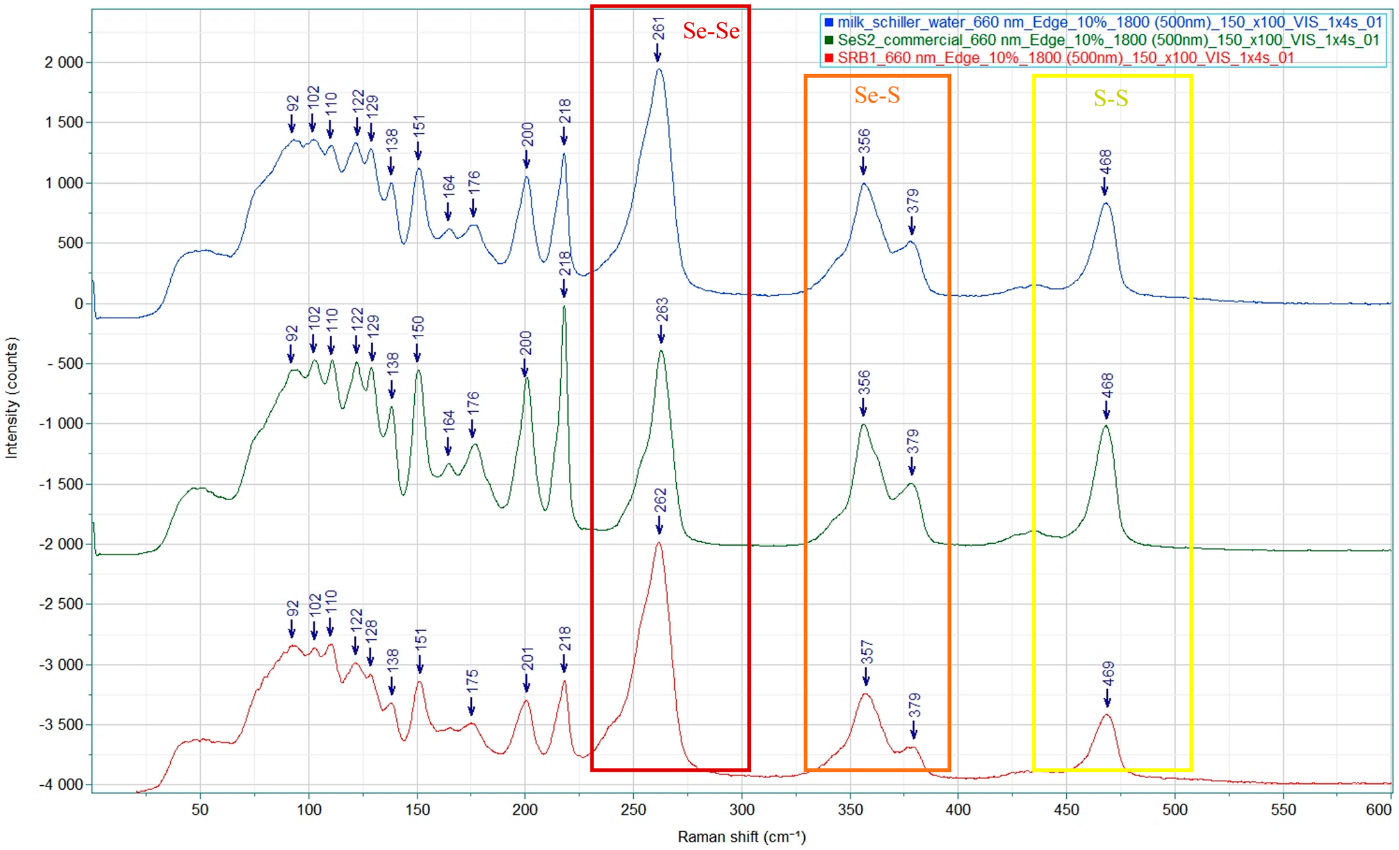

Figure S5. The comparison of the structural fingerprints of selenium sulfide produced using a mixture of spoiled milk and mineral water (A) and commercial selenium sulfide (B) as well as the standard medium mediated selenium sulfide (C).
